# Additions to the Encyrtidae and Mymaridae (Chalcidoidea) of India with new distribution and host records for some species

**DOI:** 10.3897/BDJ.3.e5216

**Published:** 2015-06-05

**Authors:** A. Rameshkumar, J. Poorani, Naveen V

**Affiliations:** ‡ Division of Insect Systematics, ICAR-National Bureau of Agricultural Insect Resources, P.B. No. 2491, HA Farm Post, Bellary Road, Hebbal, Bangalore - 560 024, India

## Abstract

**Background:**

Encyrtidae and Mymaridae of India have not been surveyed in depth and hosts are not known for most of the species as the methods of collections used are passive and do not yield firsthand information on the hosts. Based on our ongoing surveys on the Encyrtidae and Mymaridae of India, we report here new distribution and host records for some species.

**New information:**

*Acmopolynema campylurum* Xu and Lin, *Litus cynipseus* Haliday, *Omyomymar glabrum* Lin and Chiappini and *Platystethynium* Ogloblin (Mymaridae), and *Rhytidothorax purpureiscutellum* (Girault) (Encyrtidae) are reported for the first time from India. *Anagyrus aquilonaris* (Noyes and Hayat) is recorded as new to Arunachal Pradesh and Meghalaya. *Paraphaenodiscus indicus* Singh and Agarwal and *Paraphaenodiscus monawari* Bhuiya are recorded from south India for the first time, the latter on a new host, *Pulvinaria polygonata*. *Chorizococcus sorghi* Williams (Pseudococcidae) is reported as a host for *Cryptanusia ajmerensis* (Fatma & Shafee), for which no hosts are hitherto known and the male of *Cryptanusia* is documented for the first time. *Aclerda* sp. is recorded as a new host for *Neastymachus axillaris* Singh, Agarwal and Basha.

## Introduction

The Chalcidoidea fauna of India has not been systematically surveyed covering the whole country and its biodiversity hotspots like Western ghats, Eastern Himalayas and the northeastern region have been badly neglected. Though extensive faunistic studies have been carried out on encyrtids (Hayat 2006), mymarids (Subba Rao and Hayat 1985, Subba Rao and Hayat 1986) and aphelinids (Hayat 1998) in India, several descriptions are based on single specimens without taking into consideration geographical variation and not adequately illustrated. Hosts are not known for most of the known species as collections are made using passive methods like Malaise traps and yellow pan traps. We have been attempting to document the Indian species of Encyrtidae and Mymaridae with good images to facilitate their easy identification along with their hosts. We report here additions to the fauna of Indian Encyrtidae and Mymaridae and new hosts and distribution data for some species.

## Materials and methods

Extensive surveys were carried out in Ri-Bhoi, Jaintia hills, East Khasi hills, and West Khasi hills districts of Meghalaya and surrounding areas and in southern India for collection of Chalcidoidea. Different collecting methods (host rearing, net sweep, yellow pan trap and Malaise trap) were used for collection from different ecosystems. Collected parasitoids were processed and curated as per standard protocol (Noyes 1982) and identified using appropriate keys. Voucher specimens are deposited in the collections of the ICAR-National Bureau of Agricultural Insect Resources (ICAR-NBAIR), Bangalore, Karnataka, India. All measurements were done using the measurement module of Leica M205A stereo microscope and are relative. Imaging was done using Leica M205A stereo microscope and composite images were obtained from image stacks using Combine ZP. The images were touched up for clarity using Adobe Photoshop Elements 11.

## Taxon treatments

### Acmopolynema campylurum

Xu and Lin, 2002

Acmopolynema campylura Xu and Lin 2002: 147-148.

#### Materials

- **Type status:** Other material. **Occurrence:** recordedBy: A Rameshkumar; individualCount: 1; sex: female; lifeStage: Adult; **Location:** continent: Asia; country: India; stateProvince: Meghalaya; municipality: Jaintia Hills; locality: Jowai; verbatimElevation: 1297m; verbatimCoordinates: N25°27' E92°11'; **Identification:** identifiedBy: A Rameshkumar; **Event:** samplingProtocol: Net sweep; eventDate: 11-06-2013; habitat: forest land; **Record Level:** institutionID: National Bureau of Agricultural Insect Resources; institutionCode: NBAIR
- **Type status:** Other material. **Occurrence:** recordedBy: Naveen V; individualCount: 1; sex: female; lifeStage: Adult; **Location:** continent: Asia; country: India; stateProvince: Meghalaya; county: Ri-Bhoi; municipality: Umiam; locality: ICAR complex; verbatimElevation: 603m; verbatimCoordinates: N25°49', E91°52'; **Identification:** identifiedBy: A Rameshkumar; **Event:** samplingProtocol: Yellow pan traps; eventDate: 08-06-2013; habitat: weedy field; **Record Level:** institutionID: National Bureau of Agricultural Insect Resources; institutionCode: NBAIR

#### Diagnosis

**Female**. Body orange brown (Fig. 1a); antenna with F6 yellow or light brown. Vertex, scape, pronotum, coxae, femora and tibiae with short, blunt setae. Axilla with 3 or 4 foveae. Scutellum without a row of frenal foveae. Fore wing (Fig. 1c) with 2 brown spots (modified setae of types F and G on the basal spot and normal setae on the apical spot). Propodeum (Fig. 1b) with a medial groove extending from anterior margin to base of submedial carinae at posterior margin; propodeal submedial carinae very short, not reaching half length of propodeum. Ovipositor slightly exserted (Triapitsyn and Berezovskiy 2007).<br/>

#### Distribution

China, Thailand (Triapitsyn and Berezovskiy 2007), India (Meghalaya) (new record).

### Litus cynipseus

Haliday, 1833

Litus cynipseus Haliday 1833: 345.-Graham 1982: 225.

#### Materials

- **Type status:** Other material. **Occurrence:** recordedBy: A Rameshkumar; individualCount: 1; sex: female; lifeStage: Adult; **Location:** continent: Asia; country: India; stateProvince: Meghalaya; county: Ri-Bhoi; municipality: Umiam; locality: ICAR-complex; verbatimElevation: 603 m; verbatimCoordinates: N25°49' E91°52'; **Identification:** identifiedBy: A Rameshkumar; **Event:** samplingProtocol: Malaise trap; eventDate: 14-06-2013; habitat: Peach orchard; **Record Level:** institutionID: National Bureau of Agricultural Insect Resources; institutionCode: NBAIR

#### Diagnosis

**Figure 2.**
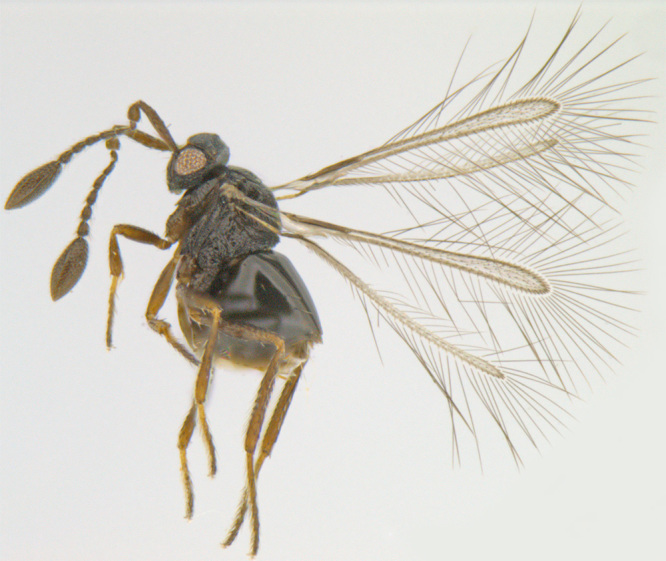
*Litus cynipseus* Haliday: Female, lateral view

**Female** (Fig. 2). Body and antenna dark brown to black, legs a little lighter (except coxae). Antenna with F1 very short, much shorter than pedicel or any other funicle segment; F2 longest, almost as long as pedicel; clava 2.2–2.5x as long as wide in lateral view, about as long as combined length of 4 preceding segments. Mesoscutum with distinct notauli; anterior part of propodeum strongly sculptured with an incomplete, often inconspicuous median longitudinal carina, posterior part of propodeum smooth. Fore wing 13–14x as long as wide, its blade slightly infumated basally and almost hyaline distally, with two rows of microtrichia along margins and many additional irregularly arranged microtrichia in distal half; longest marginal cilia 4.6–5.0x greatest width of the wing (Triapitsyn and Berezovskiy 2004).

#### Distribution

Russia, Austria, Belgium, Bulgaria, Canada, Czech Republic, Denmark, England, Finland, France, Greece, Hungary, Italy, Japan, Krygyzstan, Mexico, Moldova, Morocco, Nepal, Netherlands, Republic of Korea, Romania, Serbia and Montenegro, Slovenia, Spain, Switzerland, Turkey, USA, Wales (Triapitsyn and Berezovskiy 2004​). India (Meghalaya) (new record).

#### Biology

*Ocypus olens* (Kieffer 1913, Kryger 1950​), *Staphylinus* sp. (Staphylinidae) (Viggiani 1973​).

### Omyomymar glabrum

Lin and Chiappini, 1996

Omyomymar glabrum Lin and Chiappini 1996: 302-305.

#### Materials

- **Type status:** Other material. **Occurrence:** recordedBy: A Rameshkumar; individualCount: 1; sex: female; lifeStage: Adult; **Location:** continent: Asia; country: India; stateProvince: Meghalaya; county: Ri-Bhoi; municipality: Umiam; locality: ICAR complex; verbatimElevation: 603 m; verbatimCoordinates: N25°49' E91°52'; **Identification:** identifiedBy: A Rameshkumar; **Event:** samplingProtocol: Malaise trap; eventDate: 14-06-2013; habitat: Peach orchard; **Record Level:** institutionID: National Bureau of Agricultural Insect Resources; institutionCode: NBAIR

#### Diagnosis

**Figure 3.**
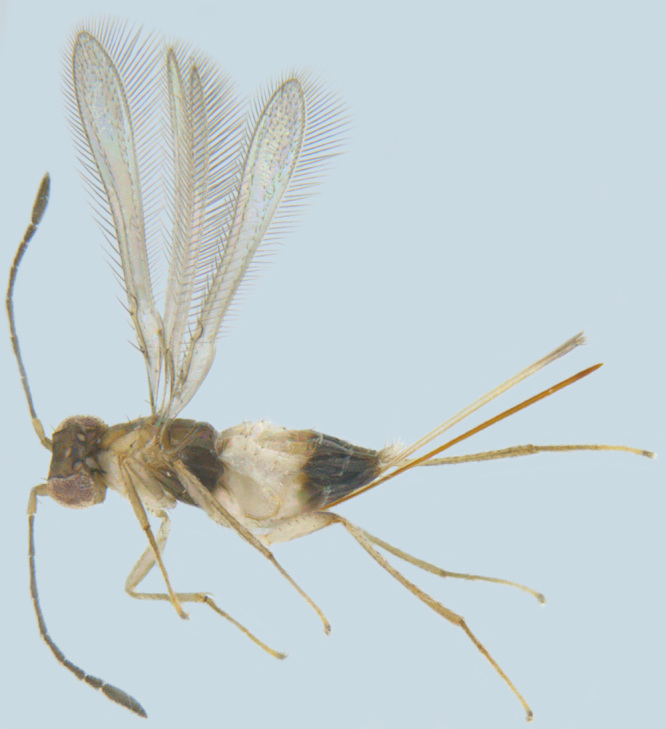
*Omyomymar glabrum* Lin and Chiappini: Female, lateral view

**Female** (Fig. 3). Body yellow, except funicle segments, club, mesopleuron, propodeum and distal half (excluding tip) of metasoma brown; eyes red. Antenna with all funicle segments cylindrical, F5 and F6 more than 2x as long as wide, F4–F6 taken together clearly longer than club, club elongate elliptical, more than 3x as long as wide, without a distinct apical digit at the tip or with a very short one; its basal segment shorter than apical one. Mesosoma a little shorter than metasoma. Fore wing about 9x as long as wide, its longest marginal cilia about 1.9x its maximum width; discal cilia irregularly scattered over apical ¼ of wing blade, anterior longitudinal line of cilia beginning beyond end of venation; hind wing almost as long as forewing, with one and a half lines discal cilia; legs slender; ovipositor longer than body length, exserted part of ovipositor at least 1.3x metasomal length (Lin and Chiappini 1996).

#### Distribution

China (Lin and Chiappini 1996​), India (Meghalaya) (new record).

### Platystethynium

Ogloblin, 1946

Platystethynium Ogloblin 1946: 290. Type species *Platystethynium onomarchicidum* Ogloblin, by original designation.

#### Materials

- **Type status:** Other material. **Occurrence:** recordedBy: A Rameshkumar; individualCount: 1; sex: female; lifeStage: Adult; **Location:** continent: Asia; country: India; stateProvince: Meghalaya; municipality: Umiam; locality: ICAR complex; verbatimElevation: 603 m; verbatimCoordinates: N25°49' E91°52'; **Identification:** identifiedBy: A Rameshkumar; **Event:** samplingProtocol: Malaise trap; eventDate: 12-06-2013; habitat: forest land; **Record Level:** institutionID: National Bureau of Agricultural Insect Resources; institutionCode: NBAIR

#### Diagnosis

**Figure 4.**
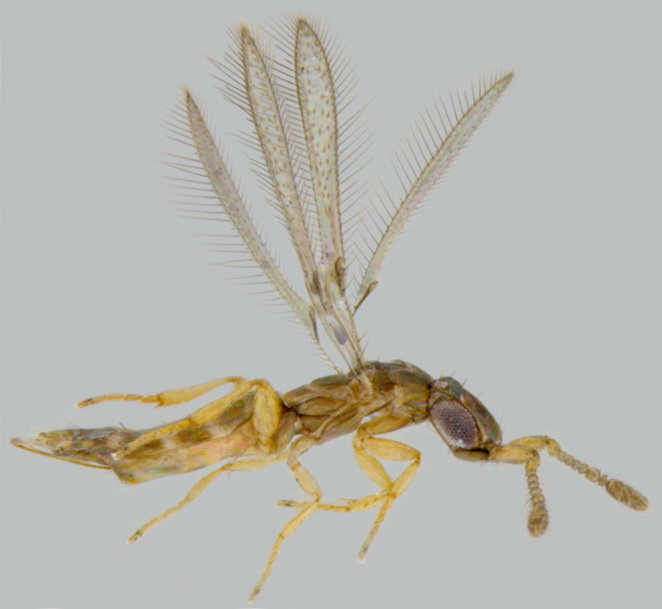
*Platystethynium* sp.: Female, lateral view

**Female** (Fig. 4). Body strongly flattened dorsoventrally. Antenna 11-segmented, with 3-segmented clava; funicle segments submoniliform; F5 and clava with placoid sensilla. Forehead distinctly divided into ventral and dorsal halves, former with two curved trabeculae, which extend from clypeus to antennal scrobes. Mandibles small, toothless, apparently not movable (without articular processes); gnathal aperture small, ventral, removed from occipital border. Epicranial sutures joining posterior ones before anterior ocellus so as to form X-shaped figure. Pronotum large, completely divided longitudinally. Wings with narrow discal blade, sharply pointed distally. Legs short and stout; fore tibiae with strong spines on ventral surface; hind femora distinctly swollen and compressed. Abdomen broadly sessile, ovipositor protruding (Ogloblin 1946​).

#### Distribution

Indonesia (Java) (Ogloblin 1946​). This constitutes the first record of this genus from India (Meghalaya).

#### Biology

**Hosts:** Eggs of Tettigonioidea, *Saltatoria* (*P. onomarchicidum* recorded from eggs of *Onomarchus uninotatus*) (Ogloblin 1946​).

### Anagyrus aquilonaris

(Noyes and Hayat, 1984)

Cremesina aquilonaris Noyes and Hayat 1984: 261-262.
Anagyrus aquilonaris : Noyes and Hayat 1994: 91-92.

#### Materials

- **Type status:** Other material. **Occurrence:** recordedBy: A Rameshkumar; individualCount: 3; sex: female; lifeStage: Adult; **Location:** continent: Asia; country: India; stateProvince: Karnataka; municipality: Bangalore; locality: Attur; verbatimElevation: 920m; verbatimCoordinates: N13°05' E77°00'; **Identification:** identifiedBy: A Rameshkumar; **Event:** samplingProtocol: Net sweep; eventDate: 26-10-2013; habitat: Weedy field; **Record Level:** institutionID: National Bureau of Agricultural Insect Resources; institutionCode: NBAIR
- **Type status:** Other material. **Occurrence:** recordedBy: A Rameshkumar; individualCount: 1; sex: female; lifeStage: Adult; **Location:** continent: Asia; country: India; stateProvince: Tamil Nadu; municipality: Thenkasi; locality: Alwarkuruchi; verbatimElevation: 160 m; verbatimCoordinates: N08°47' E77°25'; **Identification:** identifiedBy: A Rameshkumar; **Event:** samplingProtocol: Yellow pan trap; eventDate: 18-02-2015; habitat: weedy field; **Record Level:** institutionID: National Bureau of Agricultural Insect Resources; institutionCode: NBAIR
- **Type status:** Other material. **Occurrence:** recordedBy: A Rameshkumar; individualCount: 4; sex: female; lifeStage: Adult; **Location:** continent: Asia; country: India; stateProvince: Meghalaya; county: Rhi-Bhoi; municipality: Umiam; locality: ICAR complex; verbatimElevation: 603 m; verbatimCoordinates: N25°49' E91°52'; **Identification:** identifiedBy: A Rameshkumar; **Event:** samplingProtocol: Yellow pan trap; eventDate: 12-06-2013; habitat: grassy/weedy field; **Record Level:** institutionID: National Bureau of Agricultural Insect Resources; institutionCode: NBAIR
- **Type status:** Other material. **Occurrence:** recordedBy: Naveen V; individualCount: 4; sex: female; lifeStage: Adult; **Location:** continent: Asia; country: India; stateProvince: Meghalaya; county: Rhi-Bhoi; municipality: Umiam; locality: ICAR complex; verbatimElevation: 603 m; verbatimCoordinates: N25°49' E91°52'; **Identification:** identifiedBy: A Rameshkumar; **Event:** samplingProtocol: Yellow pan trap; eventDate: 10-06-2013; habitat: grassy/weedy field; **Record Level:** institutionID: National Bureau of Agricultural Insect Resources; institutionCode: NBAIR
- **Type status:** Other material. **Occurrence:** recordedBy: A Rameshkumar; individualCount: 1; sex: female; lifeStage: Adult; **Location:** continent: Asia; country: India; stateProvince: Arunachal Pradesh; county: East Siang; municipality: Pasighat; locality: College of Horticulture and Forestry campus; verbatimElevation: 153m; verbatimCoordinates: N28°07' E950; **Identification:** identifiedBy: A Rameshkumar; **Event:** samplingProtocol: Yellow pan trap; eventDate: 12-11-2014; habitat: grassy/weedy field; **Record Level:** institutionID: National Bureau of Agricultural Insect Resources; institutionCode: NBAIR

#### Diagnosis

Nominate form of *A. aquilonaris* with a characteristic reddish coloration more or less throughout dorsal side (Fig. 5a), antenna with F1, F6 and clava brown, F2-F5 generally white (Karnataka). The following variants recorded from different parts of India: head and mesosoma orange-red, metasoma dark brown, wing infuscate to a greater degree (Fig. 5b) (Tamil Nadu); head, mesosoma and metasoma deeper orange-reddish brown, antenna with F1 black, F2 white with slight infuscation, F3 pale brown, F4 black (Fig. 5c) (Arunachal Pradesh); dorsal side slightly darker reddish brown, antenna with F1 black, F2-F3 white, all the remaining segments black (Fig. 5d) (from Meghalaya).

#### Distribution

Fairly widely distributed in India (Andhra Pradesh, Assam, Bihar, Delhi, Jammu & Kashmir, Jharkhand, Karnataka, Kerala, Tamil Nadu, Uttar Pradesh, Uttarakhand (Universal Chalcidoidea Database; Hayat 2006). Arunachal Pradesh and Meghalaya (new record).

### Cryptanusia ajmerensis

(Fatma & Shafee, 1998)

Mira ajmerensis Fatma and Shafee 1988: 25.
Cryptanusia ajmerensis : Noyes and Hayat 1994: 51.

#### Materials

- **Type status:** Other material. **Occurrence:** recordedBy: Sunil Joshi; individualCount: 11 and 3; sex: females and males; lifeStage: Adult; **Location:** continent: Asia; country: India; stateProvince: Karnataka; municipality: Bangalore; locality: Doddaballapur; verbatimElevation: 880m; verbatimCoordinates: N13°13' E77°00'; **Identification:** identifiedBy: M. Hayat; **Event:** samplingProtocol: Host rearing; eventDate: 21-12-2014; **Record Level:** institutionID: National Bureau of Agricultural Insect Resources; institutionCode: NBAIR

#### Diagnosis

**Female** (Fig. 6a, b) with very prominent antenna, head and mesosoma yellowish-orange with slight infuscation, scutellum with a characteristic, heart-shaped white patch and a bunch of elongate setae before apex (Fig. 6c), metasoma black with violet metallic reflections. Antenna (Fig. 6d) with scape yellow-orange, greatly expanded, pedicel yellow, funicle dark metallic violet and spindle-shaped, clava white, basally infuscate. Head hypognathous, in front view about as long as broad (Fig. 6c). Fore wing (Fig. 6b) infuscate.

The specimens examined by us agree with the illustrations provided by Hayat 2006 (see Figs. 1588-1591) except for the presence of the scutellar spot and the setal bunch on scutellum (lost in the holotype illustrated by Hayat). The original description indicates that *C. ajmerensis* is dorsally dark brown, but apparently its colour is quite variable.

**Male** (Fig. 6f) with head yellow with a median infuscate patch; antenna brownish with elongate whorls of setae; mesosoma dark brown except scutellum orange / yellowish with a basal yellowish-white patch as in female and a few short blackish setae near apex, lacking a setal bunch; metasoma dark brown; legs yellowish; wings more or less hyaline; scape 4.6x longer than wide; funicle segments cylindrical, subequal, each about twice as long as wide, clothed with long seta; clava entire, as long as preceding two funicle segments, base of clava with 8 scale like setae; fore wing hyaline, 2.5x as long as wide; marginal vein shorter than stigmal; postmarginal vein very short; linea calva interrupted by 4 or 5 lines of seta.

#### Distribution

India: Rajasthan (Hayat 2006); new record for southern India (Karnataka).

#### Biology

Reared on *Chorizococcus sorghi* Williams (Sternorrhyncha: Pseudococcidae) infesting the roots of indeterminate plants (new host). Live adults look like small ants with vigorous wiggling of the antennae and can be readily distinguished by the characteristic antenna.

### Neastymachus axillaris

Singh, Agarwal & Basha, 1991

Neastymachus axillaris Singh et al. 1991​​: 223-224.-Hayat 2006: 164.

#### Materials

- **Type status:** Other material. **Occurrence:** recordedBy: Sunil Joshi; individualCount: 2 and 5; sex: female and male; lifeStage: Adult; **Location:** continent: Asia; country: India; stateProvince: Karnataka; municipality: Bangalore; locality: Hebbal; verbatimElevation: 920m; verbatimCoordinates: N13°02' E77°00'; **Identification:** identifiedBy: A Rameshkumar; **Event:** samplingProtocol: Host rearing; eventDate: 11-09-2014; **Record Level:** institutionID: National Bureau of Agricultural Insect Resources; institutionCode: NBAIR

#### Diagnosis

**Female** (Fig. 7a) orange-yellow, head yellow, occiput with two black patches one on either side of foramen behind each eye; antenna completely yellow; mesosoma yellow, pronotum with a wide black band across; mesoscutum with light bluish green reflection; wings hyaline; legs completely yellow; metasoma yellowish brown. Head in frontal view round, 3x as wide as frontovertex; posterior margin of mesoscutum angular and axillae produced anteriorly; fore wing 2.5x as long as wide; hind wing 4.2x as long as wide; mid-tibial spur a little longer than basitarsus. Ovipositor not exserted, more than one–quarter longer than metasoma.

**Male** (Fig. 7b) dorsally metallic green, antenna pale brown, side lobes of mesoscutum brownish, tegulae white, legs yellowish. Antennal scape about 3x as long as wide, flagellum clothed with whorls of setae; fore wing 2.6x as long as wide.

#### Biology

Reared on *Aclerda* sp. (Hemiptera: Coccoidea: Aclerdidae) on sugarcane, which constitutes a new host for this species. The specimens examined were collected from the states of Tamil Nadu and Karnataka on the same host.

### Paraphaenodiscus indicus

Singh & Agarwal, 1993

Paraphaenodiscus indicus Singh and Agarwal 1993: 25-Hayat 2006: 161.

#### Materials

- **Type status:** Other material. **Occurrence:** recordedBy: Rameshkumar A; individualCount: 1; sex: female; lifeStage: Adult; **Location:** continent: Asia; country: India; stateProvince: Karnataka; municipality: Chikkaballapura; locality: near Nandhi hills; verbatimElevation: 950m; verbatimCoordinates: N13°22' E77°00'; **Identification:** identifiedBy: J Poorani; **Event:** samplingProtocol: Yellow pan traps; eventDate: 17-09-2013; habitat: weedy field; **Record Level:** institutionID: National Bureau of Agricultural Insect Resources; institutionCode: NBAIR

#### Diagnosis

Female (Fig. 8a, b) brachypterous, dull reddish-coppery brown with metallic reflections, mesoscutum (Fig. 8c) medially metallic green, metasoma with basal tergite having metallic green reflections; antenna pale brown, F1-F3 and clava black, F4-F6 white. Scutellum (Fig. 8c) apically acutely pointed and projecting over propodeum.

#### Distribution

India: Originally described from Assam (Singh and Agarwal 1993; Hayat 2006). This constitutes the first record for southern India (Karnataka).

### Paraphaenodiscus monawari

Bhuiya, 1998

Paraphaenodiscus monawari Bhuiya 1998: 272.-Hayat 2006: 161.

#### Materials

- **Type status:** Other material. **Occurrence:** recordedBy: Sunil Joshi; individualCount: 3 and 1; sex: females and male; lifeStage: Adult; **Location:** continent: Asia; country: India; stateProvince: Karnataka; municipality: Bangalore; locality: Hebbal; verbatimElevation: 920m; verbatimCoordinates: N13°13' E77°00'; **Identification:** identifiedBy: J Poorani; **Event:** samplingProtocol: Host rearing; eventDate: 26-11-2014; **Record Level:** institutionID: National Bureau of Agricultrual Insect Resources; institutionCode: NBAIR

#### Diagnosis

**Female** (Fig. 9a) robust in outline. Head orange with greenish eyes. Mesosoma reddish brown, scutellum slightly darker and medially infuscate. Legs yellowish-orange except tarsal apices darker. Antenna with scape flattened and expanded beneath, black; F1-4 brown, F5 and F6 white, clava dark brown to black. Frontovertex less than one-fifth of head width. Fore wing infuscate.

**Male** (Fig. 9b) with dark metallic green head, mesosoma yellowish-orange, metasoma blackish except lateral sides yellowish, legs pale yellow.

#### Distribution

India: Assam (Hayat 2006​); new to southern India (Karnataka).

#### Biology

Reared from *Pulvinaria polygonata* Cockerell (Sternorrhyncha: Coccidae) (new host). Known hosts include *Pulvinaria psidii* Maskell on guava and undetermined coccids on lemon (Bhuiya 1998).

### Rhytidothorax purpureiscutellum

(Girault, 1915)

Ectromoides purpureiscutellum Girault 1915: 168.
Rhytidothorax purpureiscutellum : Noyes and Hayat 1984: 333.

#### Materials

- **Type status:** Other material. **Occurrence:** recordedBy: A Rameshkumar; individualCount: 1; sex: female; lifeStage: Adult; **Location:** continent: Asia; country: India; stateProvince: Meghalaya; municipality: Jaintia hills; locality: Jowai; verbatimElevation: 1297m; verbatimCoordinates: N25°27' E92°11'; **Identification:** identifiedBy: A Rameshkumar; **Event:** samplingProtocol: Net Sweep; eventDate: 11-06-2013; habitat: forest land; **Record Level:** institutionID: National Bureau of Agricultural Insect Resources; institutionCode: NBAIR

#### Diagnosis

**Figure 10.**
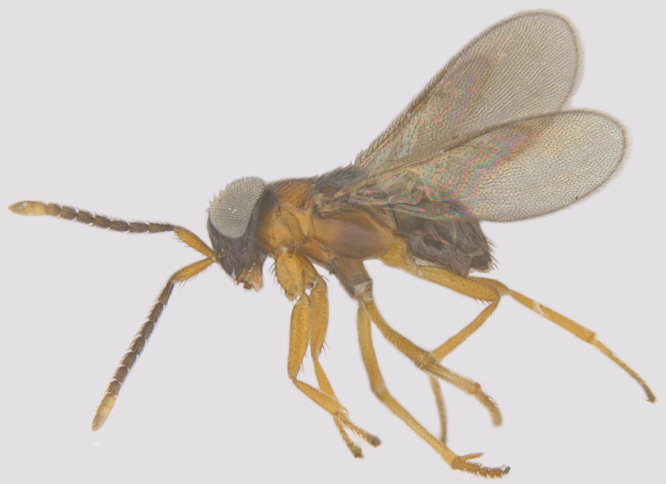
*Rhytidothorax purpureiscutellum* (Girault): Female, lateral view

**Female** (Fig. 10). Body orange yellow with varying degrees of infuscation, head, abdomen, scutellum, pronotum and cephalic part of scutum at meson narrowly metallic; wings slightly yellowish throughout; scape concolorous; funicle and pedicel purple, club white; distal two funicle segments inclined to the paler; pedicel subequal to F1, F4 and F5 subequal, a little shorter, F6 still shorter, a little longer than wide; clava no wider than funicle and not quite half its length; cheeks about half the length of eyes; frons with some punctures; scutum finely scaly, scutellum glabrous; scutum with numerous obscure setigerous punctures, scutellum with only few; axillae separated for a short distance (Girault 1915).

#### Distribution

Australia (Girault 1915). This is a new record for India (Meghalaya).

## Supplementary Material

XML Treatment for Acmopolynema campylurum

XML Treatment for Litus cynipseus

XML Treatment for Omyomymar glabrum

XML Treatment for Platystethynium

XML Treatment for Anagyrus aquilonaris

XML Treatment for Cryptanusia ajmerensis

XML Treatment for Neastymachus axillaris

XML Treatment for Paraphaenodiscus indicus

XML Treatment for Paraphaenodiscus monawari

XML Treatment for Rhytidothorax purpureiscutellum

## Figures and Tables

**Figure 1a.**
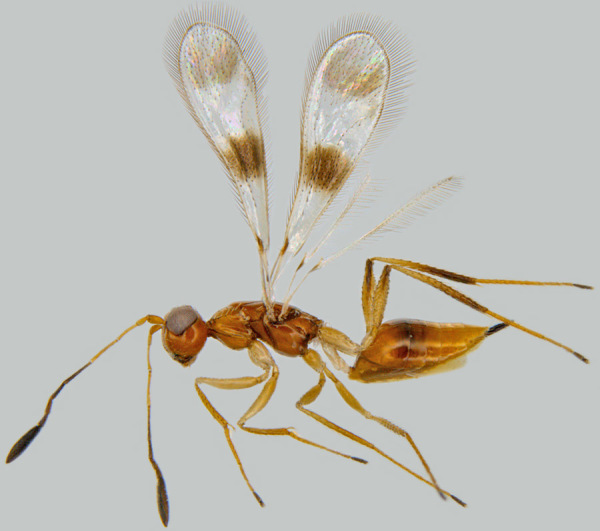
Female in profile

**Figure 1b.**
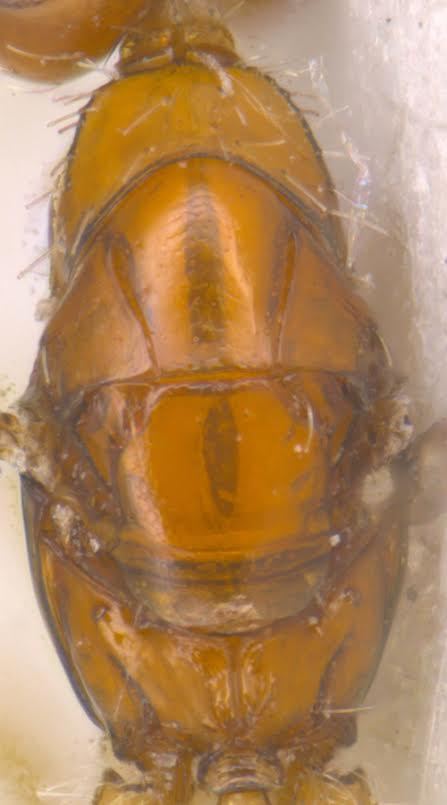
Mesosoma

**Figure 1c.**
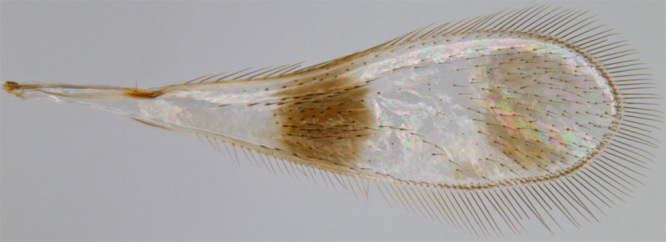
Fore wing

**Figure 5a.**
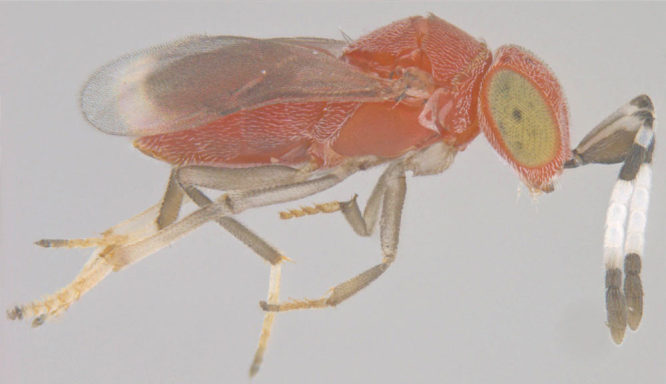
Typical form

**Figure 5b.**
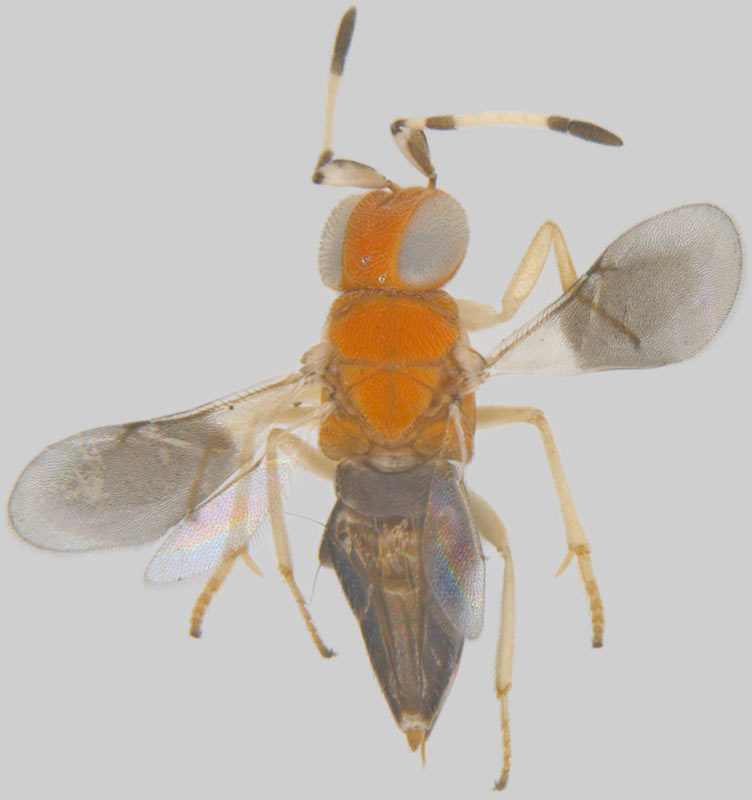
Variant with dark abdomen

**Figure 5c.**
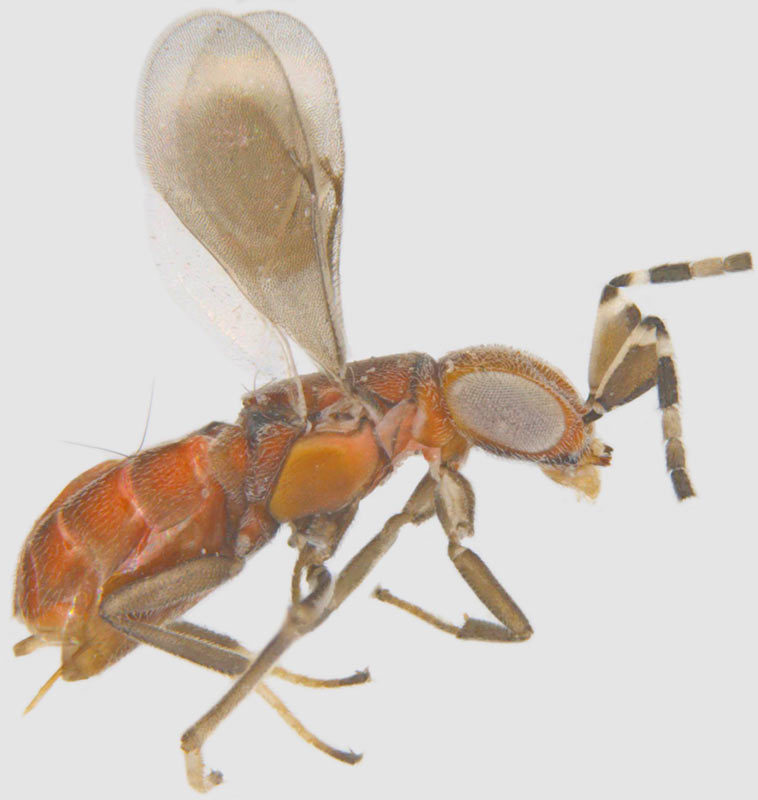
Form with variable antennal coloration

**Figure 5d.**
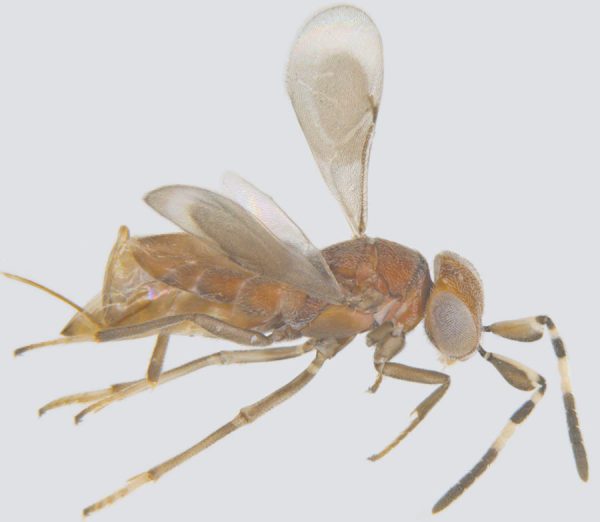
Form with variable antennal coloration

**Figure 6a.**
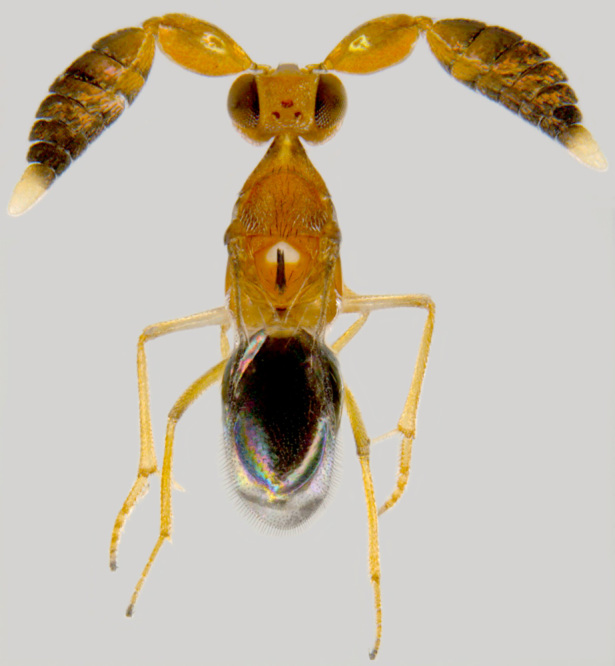
Female, dorsal view

**Figure 6b.**
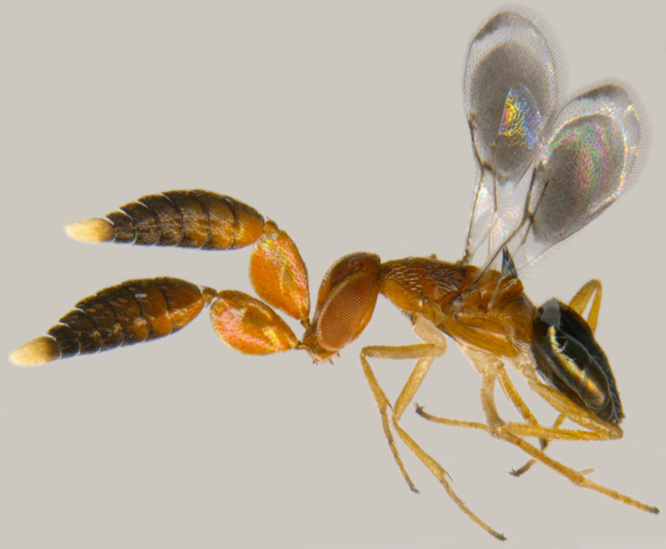
Female, lateral view

**Figure 6c.**
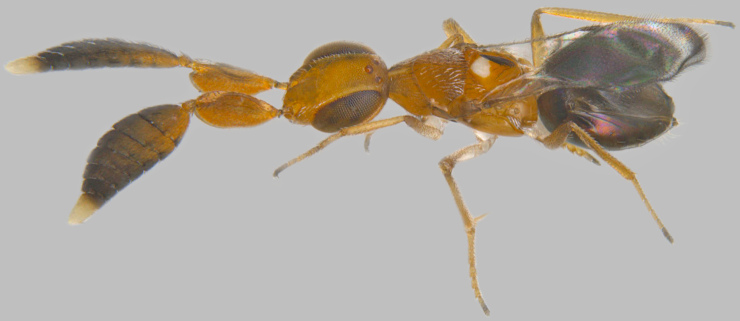
Female, dorsolateral view

**Figure 6d.**
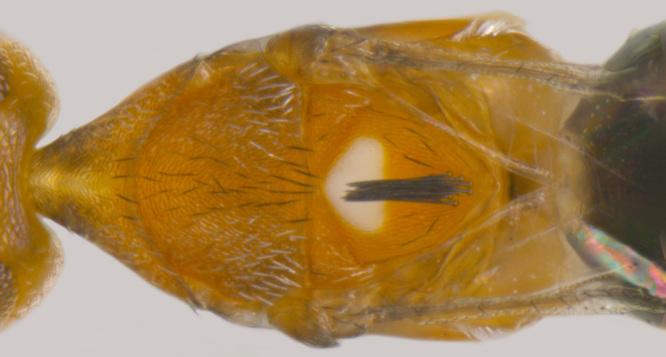
Female, mesosoma

**Figure 6e.**
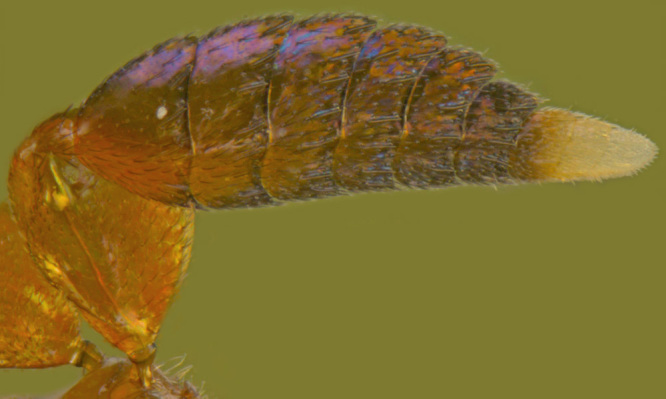
Female, antenna

**Figure 6f.**
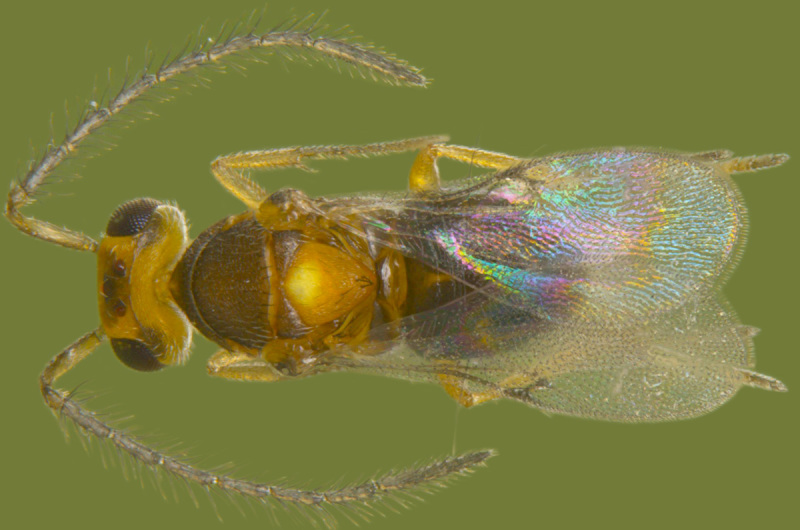
Male, dorsal view

**Figure 7a.**
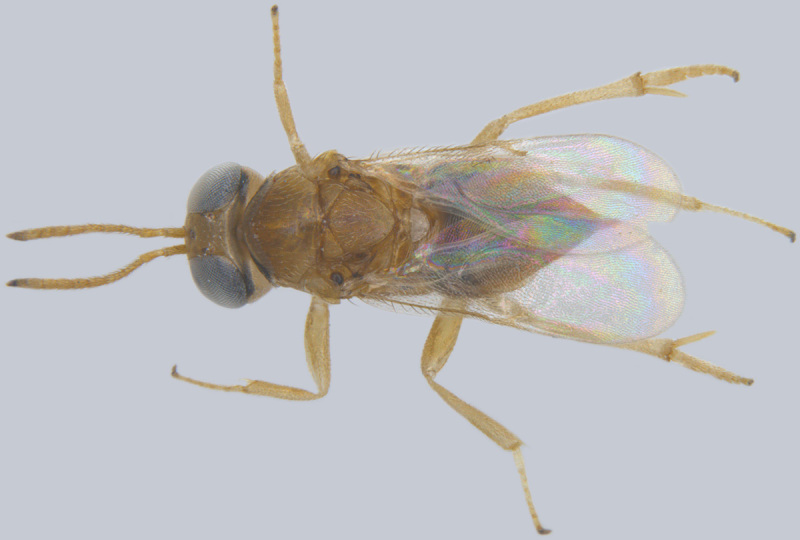
Female, dorsal view

**Figure 7b.**
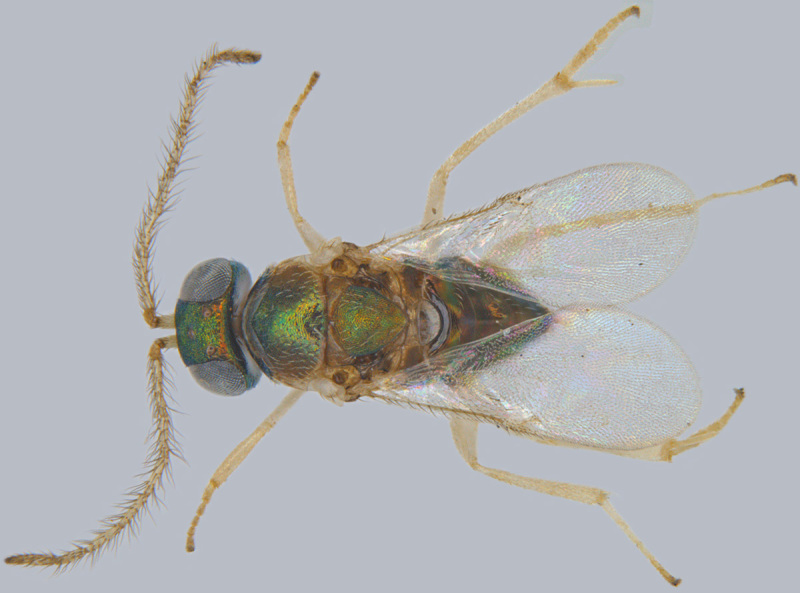
Male, dorsal view

**Figure 8a.**
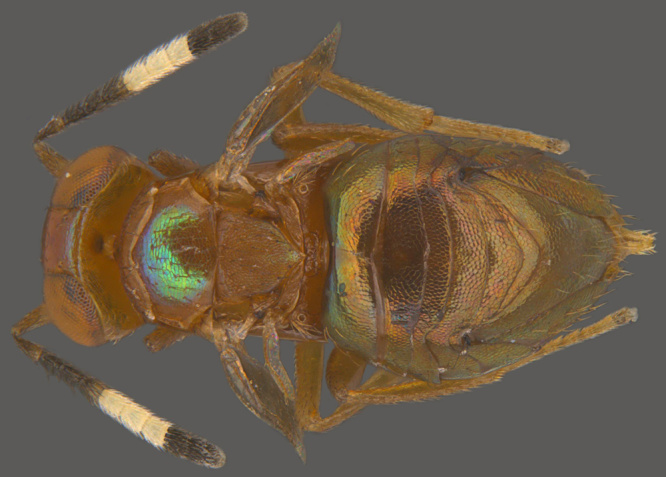
Female, dorsal view

**Figure 8b.**
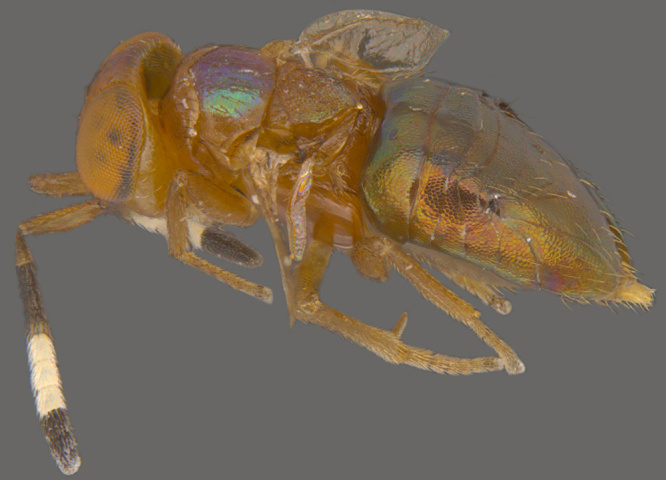
Female, lateral view

**Figure 8c.**
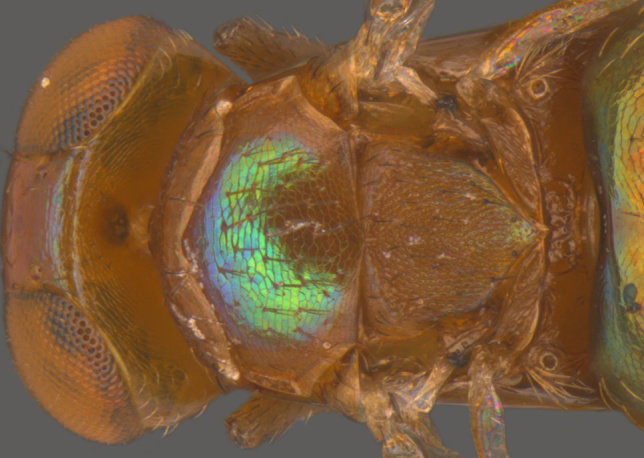
Mesosoma

**Figure 9a.**
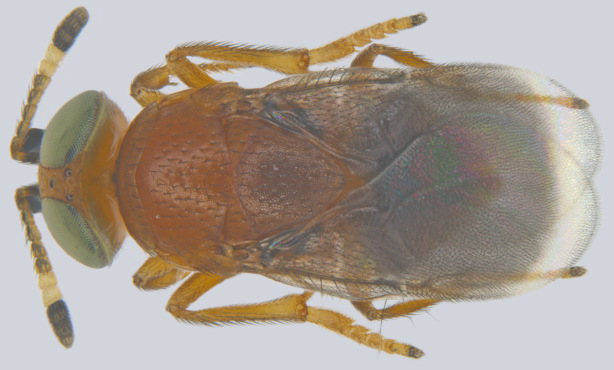
Female, dorsal view

**Figure 9b.**
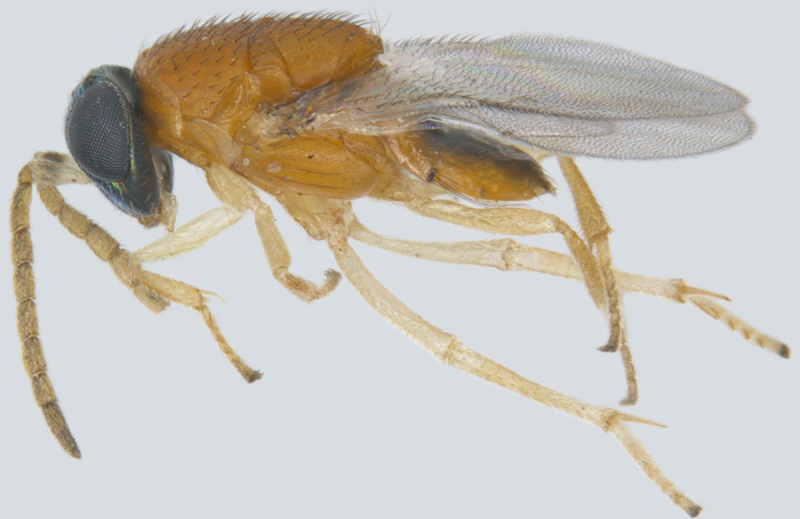
Male, lateral view
